# Mass Customization of Polylactic Acid (PLA) Parts via a Hybrid Manufacturing Process

**DOI:** 10.3390/polym14245413

**Published:** 2022-12-10

**Authors:** Ke Gong, Handai Liu, Cheng Huang, Qinyu Jiang, Han Xu, Zhi Cao, Evert Fuenmayor, Ian Major

**Affiliations:** 1 PRISM Research Institute, Technological University of Shannon: Midlands and Midwest, Athlone Campus, University Road, N37 HD68 Athlone, Ireland; 2 School of Mechanical and Electronic Engineering, East China University of Technology, No. 418 Guanglan Road, Nanchang 330013, China; 3 Faculty of Engineering & Informatics, Technological University of the Shannon: Midlands and Midwest, Athlone Campus, University Road, N37 HD68 Athlone, Ireland

**Keywords:** mass customization, hybrid manufacturing, fused deposition modelling, injection molding, overmolding, joint configuration

## Abstract

Mass customization is the development of items tailored to specific customers, but produced at low unit cost in high-volume. In this context, hybrid manufacturing (HM) combines fused deposition modeling (FDM) and injection molding (IM) to fabricate a single personalized part with minimum manufacturing cost. In this technique, inserts with different physical features are first FDM-fabricated and then IM-overmolded. This study investigated the effect of hybrid FDM-IM production technology, FDM insert geometry on mechanical properties, and micro-structural evolution of Polylactic Acid (PLA) samples. The findings indicated a comparable tensile properties of FDM-IM samples (68.38 MPa) to IM batch (68.95 MPa), emphasizing the potential of HM in the manufacturing industry. Maximum tensile stress of FDM-IM specimens shows an upward trend due to the increased infill density of preforms. In addition, overmolding interface direction results in a big gap for the maximum tensile strengths between half-length series specimens (12.99 MPa to 19.09 MPa) and half-thickness series specimens (53.83 MPa to 59.92 MPa). Furthermore, four joint configurations resulted in different mechanical performances of finished specimens, in which the female cube sample exhibits the highest tensile stress (68.38 MPa), while the batch with male T joint shows a lower value in maximum tensile strength (59.51 MPa), exhibiting a similar tensile performance with the half-thickness 75% batch without joint configuration. This study lays the groundwork for using HM to produce bespoke and mechanically improved parts over FDM alone.

## 1. Introduction

The global market for manufactured goods has become even more competitive in recent years [1]. To expedite the process of bringing products to market, many procedures related to design, testing, production, and marketing have been condensed, both in terms of time and material resources [2,3,4]. The rapid growth of the global population alongside the industrialization of society has led to several manufacturing techniques to match demand to production means. This combined, with the condensation of product development, resulted in the favoring of rapid production techniques such as injection molding (IM), which first appeared in 1870s [5].

IM is a conventional manufacturing technique which has been applied in mass production for decades. It normally starts with the mold closing, the injection of molten material into the mold cavity, and products ejected from the mold following by the material solidification [6]. Up to now, research interest has broadened the versatility of IM into distinct applications. IM offers product with superior tensile strength and stiffness when compared with compression molded ones [7]. IM offers large-scale manufacturing capability, and a diverse range of thermoplastics materials compatible with the process, thus there has been an expansion on the different applications beyond the polymer industry and daily necessities production [8]. Nevertheless, there are still some drawbacks with respect to IM, for example, warpage for the injection-molded samples, high cost in small sample run set up and part design restriction.

Additive manufacturing (AM) in its modern form made its debut in the 1980′s, when the first fully operational production system based on the use of photo-curable polymer resins for the creation of object by applying concentrated energy to a specific region of the liquid, forcing it to solidify [9]. AM is an innovative manufacturing process varying from the traditional methods as it can generate the products with minimum waste [10]. This accommodating manufacturing method consists of several techniques including stereolithography (STL) for the photopolymer liquid, fused deposition modelling (FDM) for plastic filaments, laminated object manufacturing (LOM) for the material of plastic laminations, selective laser sintering (SLS) for powder of plastic or metal and digital light processing (DLP) for thermosetting resins among others [11,12,13,14,15]. Most of these processes fabricate objects based on digital models created using computer aided design (CAD), and the multi-selection of material utilized in the manufacturing process enables AM to produce components with high flexibility [16].

Specifically to FDM technique, fabrication of specimens occurs by controlling the position of extruded molten filament and thereafter collecting onto the build platform in which layers are fused together [17]. Its low usage cost, simplicity-to-operate and multi-material capability have cemented this technique in various fields, including pharmaceutical, environment, agriculture, healthcare, automotive industry, aerospace industries, food science, research and household industry [18,19,20,21,22,23,24,25,26,27,28,29,30,31,32,33,34]. However, some limitations of FDM, include reduced mechanical strength and longer production times when compared to samples obtained via more traditional manufacturing techniques, the tight geometrical tolerance of feedstock material to name a few that continue to pose challenges for users and the adaptation of this technology [35]. In order to evaluate the effects of the building parameters’ impact upon specimens’ mechanical performances, several previous works have investigated how the blended filaments with varied weight percentage, printing velocity, nozzle diameter, orientation of building, layer thickness, raster angle, raster width, air gap, infill density, infill pattern, the rate of feeding and so on might alter these properties [36,37,38,39,40,41,42,43,44,45,46,47,48,49]. These studies showed some potential for improving the mechanical performance on FDM products and lowering the cost of small-volume goods by adjusting production settings.

The unique characteristics of polylactic acid (PLA), such as renewability, biodegradability, 4D printability and few requirements to fabricate perfect-quality samples have prompted its use in medical and tissue engineering applications [50,51,52]. However, it still undergoes some of its weaknesses, especially low impact resistance [53], which severely restricts its commercial applications. Several research studies have been conducted on PLA samples fabricated in FDM or IM alone, the performances of specimens under the FDM-IM hybrid manufacturing is still unclear.

In this study, the microstructure and mechanical properties of HM samples combining FDM and IM with varying production parameters were investigated in the pursuit of mass customization (MC). MC is a manufacturing approach in various sectors that focuses on creating uniquely customized items for the user while maintaining the economies of scale afforded by mass production. The HM used here combined FDM for the production of a substrate base and followed by IM [34], in a process known as overmolding. The test specimens in this study are those created with HM. These samples are created in two stages: 1. FDM creates preforms with varying infill density and geometry; 2. Insert these preforms into the mold cavity to begin the IM technique. All samples were tested for dimensional change, microstructure, and tensile performances to determine the effect of this HM on final part performance and quality.

## 2. Materials and Methods

### 2.1. Materials

The PLA filament (neutral color, 1.75 mm diameter) assembled in the filament reel was purchased from the Real Filament Company in Wateringen, The Netherlands. The virgin PLA 4043D biopolymer utilized in the IM process were supplied from NatureWorks Corporation in 15305 Minnetonka Blvd., MN, USA.

### 2.2. Preparation of Samples

#### 2.2.1. Details of Fabricated Samples

All manufactured samples were separated into three series in this study, the FDM, IM and HYM specimens, whose dimensions comply with the ASTM-D638-3 Standard. The fabrication of HM specimens can be divided into two stages, in which the FDM inserts were created firstly and pushing these FDM preforms into the mold cavity to proceed the overmolding process.

#### 2.2.2. Fused Deposition Modelling

The AM technique utilized in the fabrication process was FDM. The PLA filament used here was firstly dried in the oven using 60 °C for 4 h due to PLA’s moisture absorption which can adversely affect the mechanical output of fabricated samples [54], and then used in the FDM process. SolidWorks 2016 Edition Software (Dassault Systèmes, Waltham, MA, USA) was used to design the CAD models of FDM tensile dumbbell and FDM preforms/inserts and export them into the corresponding STL. Files. The STL. Files were then imported into MakerGear M2 Rev. E. (M2e) 3D printer machine (MakerGear LLC, Beachwood, OH, USA) through Simplify 3D Software (Cincinnati, OH, USA) to operate the 3D printer.

**Figure 1:**
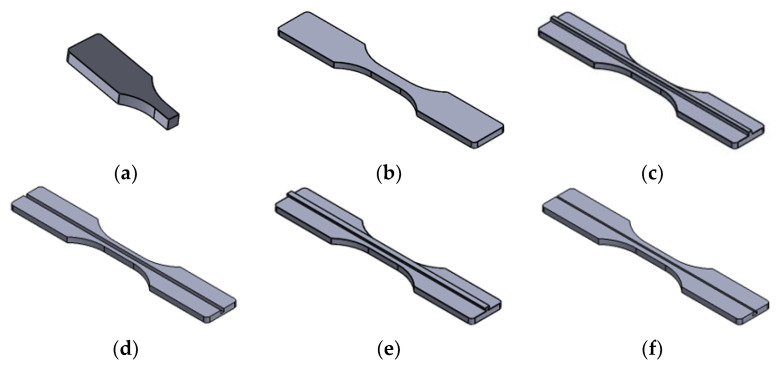
CAD Design for FDM inserts: (**a**) half-length; (**b**) half-thickness; (**c**) half-thickness male cube; (**d**) half-thickness female cube; (**e**) half-thickness male T; (**f**) half-thickness female T.

The optimal parameters utilized in the process of FDM were derived in previous publications [39,55,56,57,58,59], and kept constant in the study, these include layer thickness (0.1 mm), infill density (25%, 50%, 75%), infill orientation (45°), number of top solid layers (3), number of bottom solid layers (3), number of outline/perimeter shells (2), infill extrusion width (100%), extruder temperature (210 °C), printing bed temperature (60 °C), nozzle diameter (0.35 mm), retraction distance (1 mm), retraction speed (2400 mm/min), default printing speed (6400 mm/min). The CAD graphs for all FDM fabricated specimens are shown in Figure 1.

#### 2.2.3. Injection Molding

Injection Molding was performed on a Babyplast 6/12 injection molding machine from Rambaldi Corporation from Molteno, Italy. The temperature of plasticizing zone, chamber and nozzle were controlled and set at 185 °C, 180 °C and 170 °C, respectively. To obtain great-precision samples, the shot size was set at the same volume that is needed to fill the entire cavity [29]. Similar with the parameters used in FDM, the settings used in the IM process were determined via preliminary screening trials and are presented as follows: shot size (28 mm for HYM samples and 40 mm for IM samples), cooling time (20 s), 1st injection pressure (100 bar), 1st injection pressure time (3.5 s), 2nd injection pressure (50 bar), 2nd injection pressure time (3 s), 2nd pressure setting (3 mm), decompression (2 mm), injection speed (95%), 2nd injection speed (50%). Furthermore, the virgin PLA material utilized to IM was firstly dried in the heating oven with a temperature of 60 °C for 4 h due to the material’s tendency for moisture absorption [54], and were then applied in prior to the process of the IM process. All FDM preforms were firstly pushed into the mold and then injection molded using the PLA material to fill the cavity to produce corresponding HM samples.

### 2.3. Tensile Test

The mechanical properties of the samples based on ASTM-D638-3 were determined through the Lloyd LRX Universal tester from Lloyd Instruments Ltd., Bognor Regis in UK at a movement speed of 5 cm/min. All samples were placed in a dryer using 40 °C for a night prior to the tensile test.

In this study, a vernier caliper measured the length and thickness of gauge prior to the tensile test.

### 2.4. Macrostructure Observation

The macrostructures of all fabricated samples/cross sections of gauges for inserts were observed by a Nikon ShuttlePix P-MFSC Firmware Digital Microscope with a range of *20 magnitudes prior to the tensile tests.

### 2.5. Statistical Analysis

The data analysis was carried out through GraphPad Prism 9 (GraphPad Software Inc., San Diego, CA, USA). The data was imported into the software, in which the mean and standard deviation values were obtained from the replicated data. In addition, a one-way analysis of variance (ANOVA) with a Tuckey’s Multiple Comparison Test focusing on the joint configurations was applied here to determine the significance of the differences between each batch, where the *p*-value was set as 0.05.

### 2.6. Scanning Electron Microscopy

The scanning electron microscopy (SEM) was carried out with a Mira SEM (Tescan Oxford Instruments, Abingdon-on-Thames, UK) with a magnification of *50 to observe the surface morphology of samples after the tensile test using secondary electron. Samples tested via SEM were placed on aluminum stubs and were then gold coated with Baltec SCD 005 sputter coater (BAL-TEC GmbH, Lubeck, Germany) to mollify charging’s impact on the observation [60].

## 3. Results and Discussion

The MC philosophy separates sophisticated manufacturing processes into several distinct stages to offer customers bespoken products at reasonable costs [29,61,62]. The HM batches were produced in line with the MC theory using an FDM-IM hybrid manufacturing technique which combines the customized manufacturing from FDM and mass manufacturing ability from IM. In our manufacturing technique, batches of FDM inserts with varied joint configurations were acquired in the first stage, these inserts were then delivered to the injection molding machine for the overmolding process, where the final HM samples could be produced.

Manufacturing cost has always been a hotspot in modern industry [63]. In this study, one tensile bar needed 6 min to fabricate on average, which mainly depended on the infill density, whereas the IM technique took 1.5 min to produce two specimens, this made one FDM batch run 72 min to finish fabrication but 9 min for the IM batch for a batch consisting of 12 samples. However, the HM technique applied in this study would save cost since it took 36 min on producing inserts plus 9 min on overmolding process, with a total of 45 min for each batch. Moreover, even two inserts in different batches, could be pushed into the cavity to proceed the overmolding technique at the meantime and this would be finished under 2 min for each fabrication. These results demonstrate a significant reduction in manufacturing cost, which popularized this HM in the future manufacturing industry.

**Figure 2:**
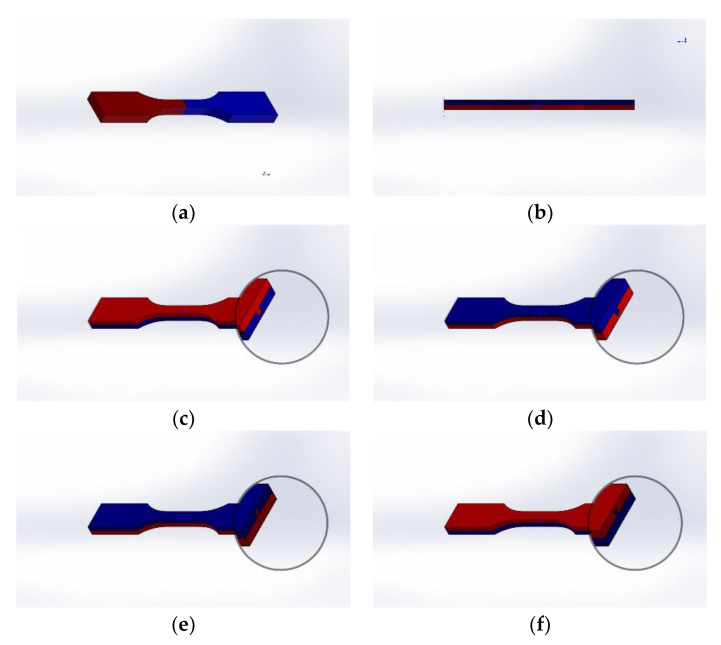
CAD design for fabricated HM samples: (**a**) half-length; (**b**) half-thickness; (**c**) half-thickness male cube; (**d**) half-thickness female cube; (**e**) half-thickness male T; (**f**) half-thickness female T. Red color represents the FDM preform and blue the IM substrate.

Figure 2 shows all batches of HM samples, where the red part is fabricated using FDM and the blue part is finished through IM. Table 1 lists all specimens fabricated in this study and these were labelled based on their individual manufacturing routes and parameters.

### 3.1. Macrostructure Observation for All Fabricated Samples

Table 2 shows the results of physical observation for all fabricated samples, which are subjected to the ASTM guidelines. In addition, the lower standard deviations of dimensions in HYM samples indicate FDM’s poor dimension control can be improved via IM in overmolding here, highlighting the potential benefit of HM on enhancing the accuracy of finished products.

### 3.2. Tensile Performances for All Fabricated Samples

To date, several prior studies concluded that FDM-fabricated samples would exhibit worse mechanical performance compared to injection-molded samples, particularly in the tensile test [64,65,66]. In this context, the authors suggest a comparison of the tensile performances of PLA material produced by FDM, IM and FDM-IM hybrid manufacturing to emphasize the variation in tensile performance due to the technique applied. The comparison is vital for realizing the full potential of HM products for improvement of FDM and IM goods in some applications.

The test results, including their average values and standard deviations, of the maximum tensile stress (σ) and Young’s Modulus (E) for FDM and IM samples are tabulated in Table 3 and for HM samples in Table 4.

The results of all batches of samples ranged as tensile strength went from lowest to highest, σ = [12.99, 68.95] MPa respectively., Young’s Modulus (E) on the other hand range went from 612.32 MPa to 1130.07 MPa (E = [612.32, 1130.07] MPa) due to their varied parameter settings, emphasizing the possibility of mechanical performance and customization possibilities for applications where these performances would be required for parts performance. In addition, larger standard deviations for the maximum tensile stress and Young’s Moduli can be found in HL-NJ series than HT-NJ series due to the certain manufacturing condition, which agrees with the findings of the effects on mechanical performance based on the variations of building orientations and infill angles for FDM manufactured samples in previous studies [41,43,44,67]. This indicates a higher reliability in industry for HT series compared to the HL ones, increasing opportunities for HT series employed in the future appliances.

**Figure 3:**
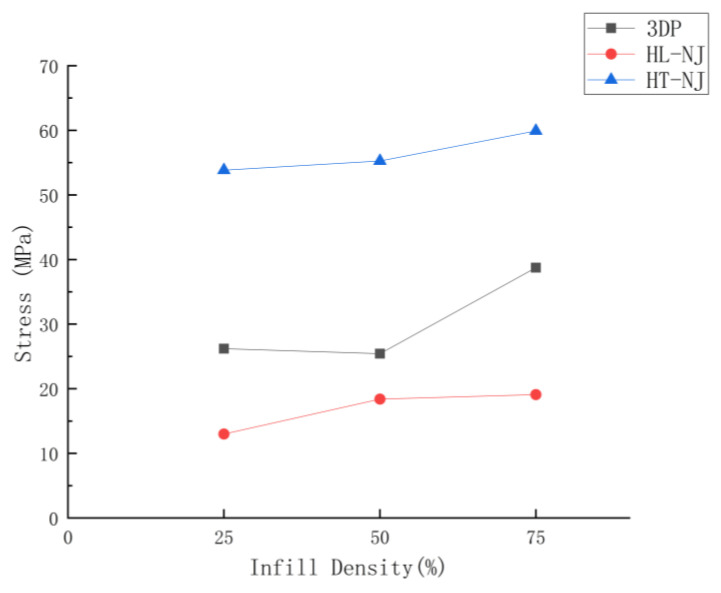
Graphical comparison of average stress value for the tensile samples as a function of infill density.

**Figure 4:**
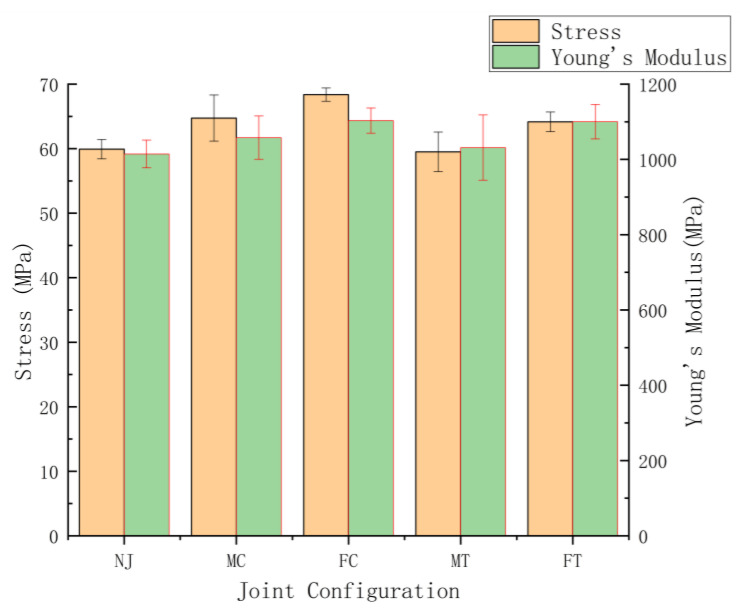
Graphical comparison of average stress value and Young’s Modulus for the HT series samples (75% infill density) as a function of joint configuration.

To determine the correlation between the parameters applied in the study on the tensile performance, below there are two graphical representations based on the tensile results displayed in Figure 3 and Figure 4. These two figures show the mechanical performances as a function of infill density and joint configurations.

The effects of parameters applied in the HM technique on the tensile performance of PLA samples are determined in the following sections.

#### 3.2.1. Effects of Manufacturing Technique on the Mechanical Performance

**Figure 5:**
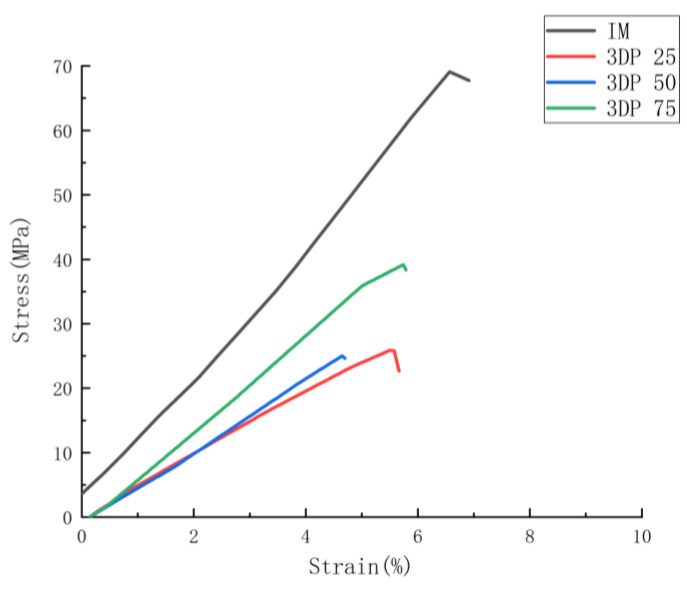
Average stress-strain curves for IM and FDM samples.

The tensile performances of one IM batch and three batches of FDM samples (IM, FDM 25, FDM 50 and FDM 75) were compared in Table 3 and Figure 5. Firstly, IM batch displays the best mechanical performance amongst these four batches for tested properties (68.95 MPa for maximum tensile stress and 1130.07 MPa for Young’s Modulus). As for the FDM samples, the FDM 75 shows the greatest tensile performance in the group (38.75 MPa in maximum stress and 814.3 MPa for Young’s Modulus), but still finds its poor performance if compared with the IM one (38.75 MPa < 68.95 MPa in tensile stress and 814.3 MPa < 1130.07 MPa). It can be found that the IM samples show better tensile performance compared with the FDM ones, which is caused of the good-quality polymer chain entanglement produced in IM processing and higher material density produced by the pressures used during manufacturing [35,65].

To figure out the effect of HM on the mechanical performance, two types of HM samples were taken into account in this study: the half-length samples which were overmolded at a single mid-point along the tensile bar (HL-NJ 25, HL-NJ 50 and HL-NJ 75) and half-thickness samples (HT-NJ series, MC, FC, MT and FT) which were overmolded along the full length of the FDM inserts. The tensile results for HM samples displayed in Table 4 found a gap between half-length series exhibiting tensile stress from 12.99 MPa to 19.09 MPa with half-thickness series ranging from 53.83 MPa to 68.38 MPa. This enhanced tensile performance from FDM batches to half-thickness series indicates the potential applicability of this HM technique in the future manufacturing industry, especially those requesting outstanding mechanical performances, such as heart stents utilized in the medical field and flexible packaging film in delivery field.

#### 3.2.2. Effects of Infill Density on the Mechanical Performance

Infill density refers to the quantity of material utilized to fill the interior region of a layer [68], which is a critical parameter applied in FDM technique since it affects manufacturing time and mechanical performance [69]. Hence, infill density is considered in this study to determine its effect in HM samples.

A first glance at the effect of infill density in the FDM samples (Table 3, Figure 3 and Figure 5), where we found similar performances in FDM 25 and FDM 50 batches (26.21 MPa and 25.43 MPa for maximum tensile stress and 612.32 MPa along with 639.33 MPa for Young’s Modulus), but a clear increasing phenomenon can be observed if infill density sets at 75% (38.75 MPa for maximum tensile stress and 814.30 MPa for Young’s Modulus). This performance transition can be explained by the high-density structure in FDM 75 batch, which provides more material to withstand the loading and thus superior mechanical performance than FDM 25 and FDM 50 batches.

**Figure 6:**
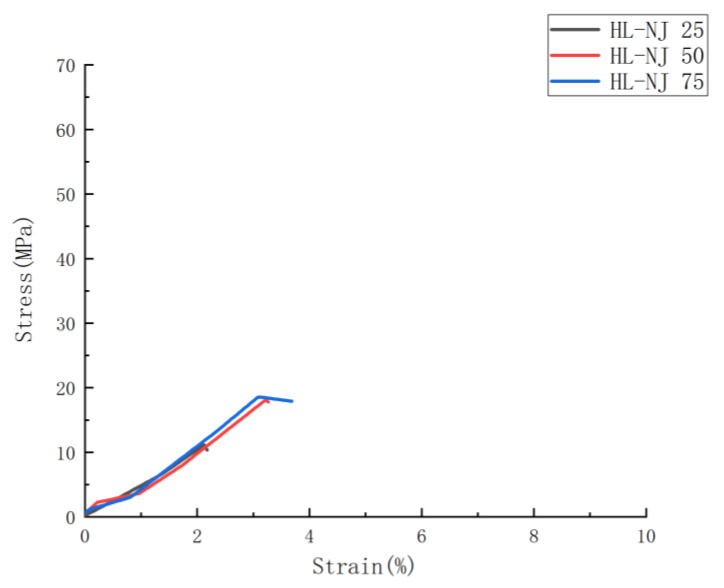
Average stress-strain curves for HL-NJ series in function of infill density.

**Figure 7:**
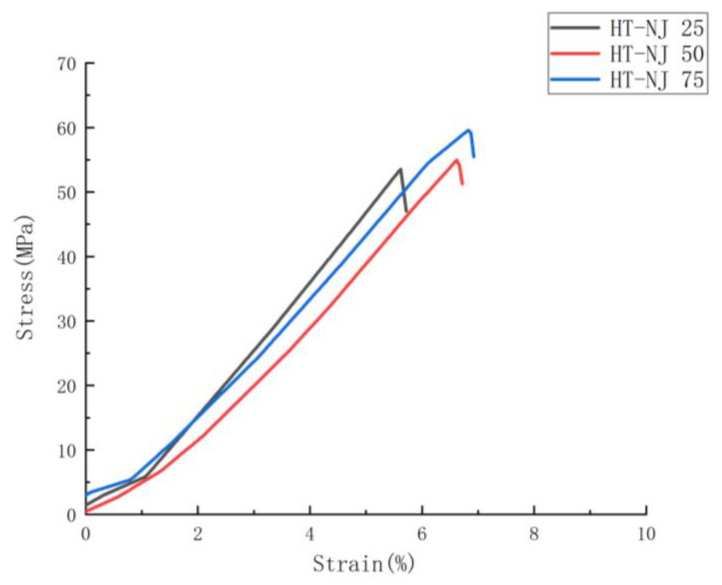
Average stress-strain curves for HT-NJ series in function of infill density.

**Figure 8:**
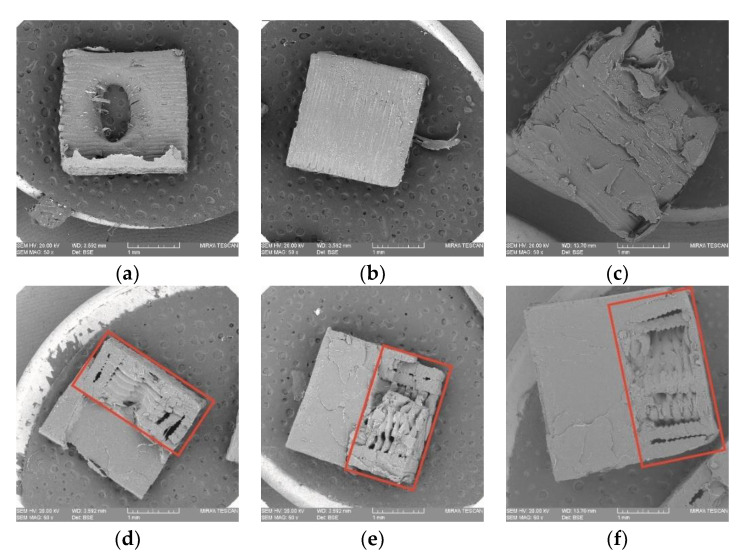
SEM observations on the fractured surfaces of (**a**) HL-NJ 25; (**b**) HL-NJ 50; (**c**) HL-NJ 75; (**d**) HT-NJ 25; (**e**) HT-NJ 50; (**f**) HT-NJ 75, in which the FDM preforms have been contoured using red square for HT series.

Figure 3, Figure 5, Figure 6 and Figure 7 display the tensile stress as a function of infill density in FDM, HL-NJ and HT-NJ samples, where we found the same trend in HL-NJ and HT-NJ that the higher infill density contributed to an increasing tensile stress. For the HM samples, the FDM inserts with higher infill density were provided better internal structure than those with lower infill. The higher-infill inserts, such as (a–c) and (d–f) shown in Figure 8 could produce stronger molecular bonding compared with those inserts with lower infill density, resulting in better joining between FDM inserts and injection-molded polymer. 

Observed in Figure 8a, is a phenomenon that is which should be thoroughly considered in the HL-NJ 25 manufacturing process is the damage to the FDM insert caused by injection-molded material; this is another indicator of worse mechanical performance in specimens with lower infill density since this insert at this joint configuration and infill density could not withstand the compressive forces of the injection molding volume during manufacturing. Meanwhile, it could be clearly seen from the SEM images in Figure 8a–c that FDM technique had resulted in void and uneven distribution of molten polymer for the internal structure of FDM area during the 3D printing process, drawing low inter laminar adhesion for FDM parts, which supported that the lower-infill density FDM samples provided worse mechanical properties.

In short, infill density shows its crucial impact in tensile performance (stress, stiffness and deformation at fracture). Thus, the high infill density (75%) exhibits the greatest mechanical performance in the tensile stress for FDM-75, HL-NJ 75 and HT-NJ 75, respectively. In addition, we can figure out a ductile behavior in HL-NJ 75 and HT-NJ 75 if compared with the ones with 25% and 50% infill density in Figure 8. This indicates a varied mechanical performance of HM specimen can be resulted from a bespoken characteristics (infill density) in the manufacturing industry. In addition, the low deviations between the tensile strengths of batches in FDM 50 and FDM 75 or HL-NJ 50 and HL-NJ 75 demonstrate a proper analysis of the demands should be conducted prior to the production stage, which will reduce the manufacturing cost with limited material waste.

#### 3.2.3. Effects of Interface Direction on the Mechanical Performance

A large tensile result gap can be found between the half-length samples with the half-thickness samples, indicating two series of FDM inserts played anisotropic roles in the mechanical performances (maximum tensile stress) of HM samples. Three batches of HT-NJ showed their greater mechanical performances in comparison with the HL-NJ 25 to 75, approximately from 76% (HT-NJ 25 with HL-NJ 25) down to 66.7% (HT-NJ 50 with HL-NJ 25) for maximum tensile stress, which can be found in Table 4, Figure 6 and Figure 7. The results of stiffness were in line with the value related to the maximum tensile stress as well, that the Young’s Modulus values for half thickness series are larger than the ones for half-length series, ranging from 24.9% (HT-NJ 75 with HL-NJ 75) to 37.5% (HT-NJ 25 with HL-NJ 25).

This kind of phenomenon can be explained through the failure modes in the tensile test: inter-layer failure and trans-layer failure, which agreed with some previous pieces of literature related to the building orientations for FDM samples [36,37,41,44,63]. For the half-length series samples, the tensile load was loaded perpendicular to the interface, leading to an inter-layer failure. In these cases, interfaces between the inserts and overmolded parts withstood most tensile loadings, leading to the low strain values in Figure 6 and brittle behaviors in Figure 8a–c. However, the samples showed an opposite reaction in HT-NJ series, the interface directions were parallel to the tensile loading for these samples, resulting in a trans-layer failure. Figure 8d–f found several breakages since the materials were applied to withstand the tensile loading. Meanwhile, ductile behaviors were found in HT-NJ series since some greater strain values were exhibited in Figure 7 for these specimens (5–7%) than the HL-NJ ones (2–3.5%) which shown in Figure 6. It can be expected that the HT-NJ series will display a poor tensile performance if these samples were pulled in manufacturing direction [63]. Finally, Table 3 and Table 4 found larger standard deviations in HL-NJ 25, 50 and 75 than HT-NJ 25, 50 and 75, which indicate a greater function of flexibility in half thickness series samples [70].

These results underscored that the interface direction had a significant role in affecting tensile performance for HYM samples and this triggered to a superior tensile performance in HT series samples. However, a half-length series sample can be more popular in market due to the direct availability of customer-tailored features, even if its mechanical performance is worse to that of half-thickness series.

#### 3.2.4. Effects of Joint Configuration on the Mechanical Performance

**Figure 9:**
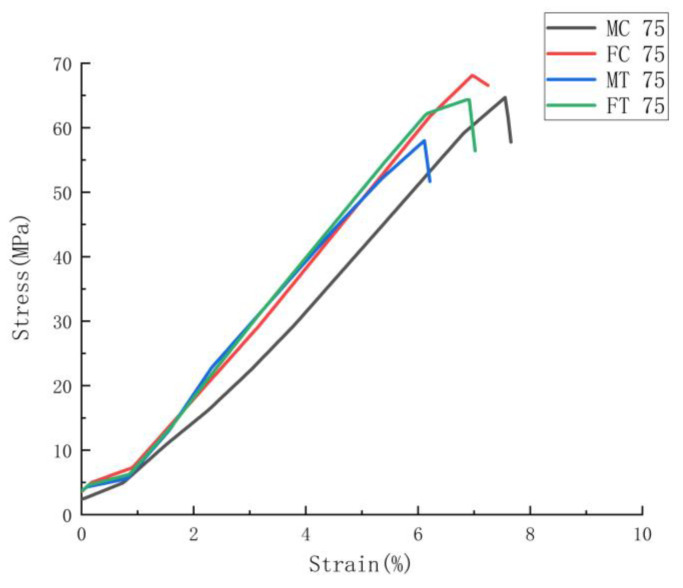
Average stress-strain curves for HT series samples with joint configurations.

**Figure 10:**
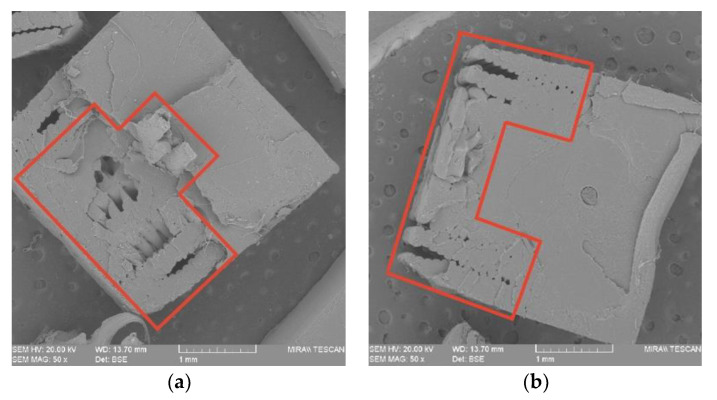

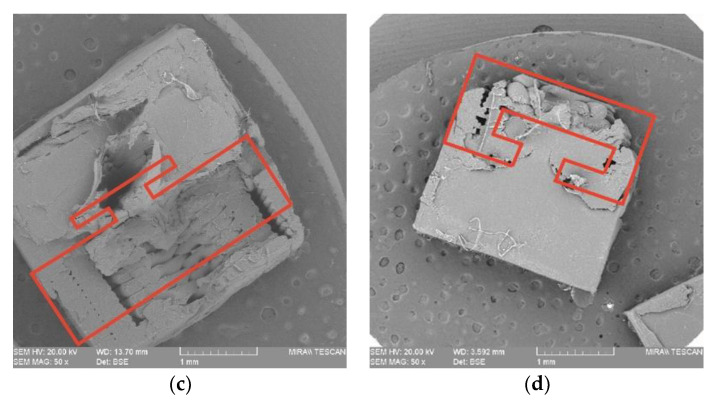
SEM observations on the fractured surfaces of (**a**) MC 75; (**b**) FC 75; (**c**) MT 75 and (**d**) FT 75, in which the FDM preforms have been indicated in red.

According to Figure 4 and Figure 9 and Table 4, the maximum average tensile stress values for HT series samples with joint configuration (MC, FC, MT and FT) can reach to 68.38 MPa (FC 75), which is nearly equal with that for injection molded batch (68.95 MPa). When compared to all batches of HT-NJ series, MT 75, the batch with the lowest tensile stress in HT series having joint configurations, nevertheless exhibits an exceptional stress gap, indicating a significant impact of joint configuration on tensile performance. The stress-strain curves shown in Figure 9 revealed that FC 75 and FT 75 had a greater tensile stress than MC 75 and MT 75. Additionally, in the tensile test, the fracture began when the tensile loading was applied but was prevented by the overmolded form, which had superior mechanical properties to the FDM preforms, as shown in Figure 10. Later, the on-going pull was affected due to the formations of new cracks, aiming to surmount the restriction from the overmolded construction. Additional cracks were then generated by increasing the tensile tension. Finally, the tear steps were produced by repeating the preceding processes.

Moreover, an interesting phenomenon can be found from Table 4, Figure 4 and Figure 9 that female joint configuration exhibits a higher tensile stress if compared with male counterparts (68.38 MPa > 64.74 MPa in cube shape and 64.16 MPa > 59.51 MPa in T shape) along with lower standard deviations, this type of result can be explained through two factors: 1. female joint configuration would offer stronger mechanical interlock in the corner of interlayers for the overmolding process; 2. more molten polymer could fill the female joints due the larger surface area available. In addition, the cube-shape joint configuration was found to have higher tensile stress and Young’s Modulus values but lower standard deviations than the T-shape joint configuration, which is a result of the PLA material’s low ductility, making it difficult to fully bond and thus allowing the stress concentration to be determined. This disagreement with the higher tensile performance of ABS specimen with T-shape joint configuration demonstrates that the material used in this HM process will affect the tensile qualities of specimens [34]. Even while the HT series samples exhibit somewhat worse mechanical performance than the IM batch, their outstanding ability in high design flexibility from FDM technique to allow for personalization make them of potential interest for future manufacturing industry, such as some unique features manufactured on the surface of laptops and coffee machine in the context of customer demands.

### 3.3. Statistical Analysis

The results of ANOVA shown in Table 5 determined the degree of freedom, sum of squares, mean square, F-ratio and *p*-value for tensile stress and found the varied joint configurations were statistically significant with the *p*-values < 0.05 for tensile stress. Moreover, the results of Tukey Test displayed in Table 6 indicated 4 levels of significant effect based on joint configurations while the comparison of NJ 75 with MT 75 and MC 75 with FT 75 showed no significant effect on tensile stress values. These statements are in line with the tensile performances shown in Table 6 and make it clear that the selection of joint configurations show a crucial effect in mechanical properties.

## 4. Conclusions

In this study, 14 batches of samples (10 batches of HM, 3 batches of FDM, and 1 batch of IM samples) were manufactured and tested. Half-length series samples that were overmolded at a single mid-point along the tensile sample, and half-thickness series samples that occurred along the entire length of the substrate were evaluated. Mechanical performance for all samples was determined using dimensional accuracy, tensile testing, and fractographic analysis to determine the effect of FDM-IM hybrid manufacturing on the final samples.

The tensile test revealed that the IM batch had the best mechanical properties of all batches. In the study, the half-thickness series samples, particularly FC 75, demonstrated comparable tensile strength and stiffness to the the IM samples. FDM samples had better mechanical properties than half-length series samples but worse than half-thickness series batches. The outstanding mechanical performance of half-thickness series samples, as well as their highly customized character, have demonstrated their advantages here, and these will do more in some everyday products with greater choice for the consumer.

The SEM revealed that half-length series samples were fractured in their joining surfaces, whereas half-thickness series samples were first broken in the joining areas and then cracks diffused to the brittle areas, resulting in the fracture. The tensile performance of the HM samples could be explained by the following statements: (1) the direction of the interface and tensile loading would significantly affect the tensile behavior (parallel direction is recommended); (2) voids located in the joining of HM samples reduce mechanical strength; (3) crack inhibition during tensile loading was brought by the over-molded polymer in HM samples with joint configurations, and this provided FC 75 with the best tensile behavior; (4) the bonding condition varies the final mechanical performance (cube shape is recommended).

However, the current study does not include any analysis of some HM parameters, such as injection molding pressure, nozzle temperature, and print speed in the FDM technique. Furthermore, more mechanical tests, such as impact and flexural tests, should be performed on these HM specimens in order to investigate mechanical performance metrics other than tensile. This catalog of PLA HM samples and their mechanical properties will aid in identifying industries and products that currently lack customization, and where this technique could be used to achieve a more complete integration of MC philosophies offered by this unique manufacturing strategy.

## Figures and Tables

**Table 1:** The range of fabricated specimens.

Batch Name	Type of Samples/Inserts	Infill Density
FDM 25	FDM Sample	25%
FDM 50	FDM Sample	50%
FDM 75	FDM Sample	75%
IM	IM Sample	——
HL-NJ 25	Half of Length (No Joint Configuration)	25%
HL-NJ 50	Half of Length (No Joint Configuration)	50%
HL-NJ 75	Half of Length (No Joint Configuration)	75%
HT-NJ 25	Half of Thickness (No Joint Configuration)	25%
HT-NJ 50	Half of Thickness (No Joint Configuration)	50%
HT-NJ 75	Half of Thickness (No Joint Configuration)	75%
MC 75	Half of Thickness Male Cube	75%
FC 75	Half of Thickness Female Cube	75%
MT 75	Half of Thickness Male T	75%
FT 75	Half of Thickness Female T	75%

**Table 2:** Dimensions of all fabricated samples.

Parameter	IM Specimens	FDM Specimens	HYM Specimens
Fillet Radius R(mm)	12.7	12.7	12.7
Thickness (mm)	3.2 ± 0.1	3.2 ± 0.2	3.2 ± 0.1
Total length (mm)	64 ± 0.1	64 ± 0.3	64 ± 0.2
Width of joint for injection and FDM (mm)	——	——	3.3 ± 0.2
Width at two ends (mm)	10 ± 0.2	10 ± 0.2	10 ± 0.2

**Table 3:** Average tensile test results (tensile stress and Young’s Modulus) and their standard deviations for IM and FDM batches.

Batch Reference	Tensile Stress, σ (MPa)	Young’s Modulus, E (MPa)
IM	68.95 ± 1.75	1130.07 ± 18.93
FDM 25	26.21 ± 1.03	612.32 ± 31.80
FDM 50	25.43 ± 2.15	639.33 ± 30.58
FDM 75	38.75 ± 1.92	814.30 ± 39.52

**Table 4:** Average tensile test results (tensile stress and Young’s Modulus) and their standard deviations for all HM batches.

Batch Reference	Tensile Stress, σ (MPa)	Young’s Modulus, E (MPa)
HL-NJ 25	12.99 ± 5.78	640.88 ± 50.43
HL-NJ 50	18.41 ± 2.96	720.85 ± 54.68
HL-NJ 75	19.09 ± 7.14	761.48 ± 71.45
HT-NJ 25	53.83 ± 2.96	1025.69 ± 45.29
HT-NJ 50	55.26 ± 2.98	984.15 ± 31.96
HT-NJ 75	59.92 ± 1.51	1020.13 ± 33.88
MC 75	64.74 ± 3.57	1057.92 ± 57.45
FC 75	68.38 ± 1.03	1103.13 ± 33.28
MT 75	59.51 ± 3.07	1031.50 ± 86.71
FT 75	64.16 ± 1.53	1100.29 ± 45.77

**Table 5:** Analysis of variance (ANOVA) of model for tensile stress. Degree of freedom (DF); sum of squares (SS); mean square (MS); F-ratio (F); *p*-value (P).

Source	DF	Ultimate Tensile Stress			
		SS	MS	F	P
Model	4	544	135.998	21.98	0
Residual	45	278.5	6.189		
Total	49	822.5			

**Table 6:** Results of Tukey Test for individual batches, in which **** indicates the level-4 significance, *** indicates a level-3 significance, ** indicates a level-2 significance, * indicates a level-2 significance, and ns indicates no significance.

Tukey’s Multiple Comparisons Test	Mean Diff.	95.00% CI of Diff.	Summary	Adjusted *p* Value
NJ75 vs. MC75	−4.82	−7.981–−1.659	***	0.0007
NJ75 vs. FC75	−8.46	−11.62–−5.299	****	<0.0001
NJ75 vs. MT75	0.41	−2.751–3.571	ns	0.9959
NJ75 vs. FT75	−4.24	−7.401–−1.079	**	0.0036
MC75 vs. FC75	−3.64	−6.801–−0.4788	*	0.0167
MC75 vs. MT75	5.23	2.069–8.391	***	0.0002
MC75 vs. FT75	0.58	−2.581–3.741	ns	0.9847
FC75 vs. MT75	8.87	5.709–12.03	****	<0.0001
FC75 vs. FT75	4.22	1.059–7.381	**	0.0038
MT75 vs. FT75	−4.65	−7.811–−1.489	**	0.0012
